# How often are ecosystems top‐down controlled? Experiments in grassland, grasshopper, and bird systems over time and space

**DOI:** 10.1002/ecs2.70066

**Published:** 2024-11-21

**Authors:** Gary E. Belovsky, Jennifer B. Slade

**Affiliations:** ^1^ Department of Biological Sciences University of Notre Dame Notre Dame Indiana USA

**Keywords:** birds, grasshoppers, plants, spiders, trophic systems

## Abstract

Ecosystems are frequently considered to be controlled by predation (top‐down). Experiments examined this in four bird/spider/grasshopper/prairie habitats over 34 years, employing in each habitat three 100 m^2^ bird exclosures and controls (121 habitat/year cases) where plant, grasshopper, and spider abundances were measured. Top‐down control (plants decrease and grasshoppers increase with bird exclusion) was observed in only 13.2% of cases, while plants increased and grasshoppers decreased in 33.1% of cases, plants decreased and grasshoppers decreased in 25.6% of cases, and plants increased and grasshoppers increased in 28.1% of cases. Therefore, top‐down control was not common and system responses were not constant, but varied among sites, years, and directionally over time with climate change. This diversity of responses is expected given the variety of underlying processes in complex ecosystems. For example, decision tree/discriminant analysis found that plant decreases and increases with bird exclusion were correctly identified in 78.3% of cases by grasshopper hatchling abundance, plant cover, and annual net primary production (ANPP), while grasshopper decreases and increases with bird exclusion were correctly identified in 76.7% of cases by edible plant biomass per grasshopper hatchling, grasshopper hatchling abundance, and large grasshopper abundance. Analysis of other system‐wide terrestrial trophic experiments indicates that the variety of responses observed by us over time and space may be common so that system‐wide trophic responses may, in general, be more variable than either top‐down or bottom‐up as often considered.

## INTRODUCTION

Many ecologists (e.g., Terborgh & Estes, 2010), and the public (Flores, 2022), claim that predation controls ecosystems, a top‐down (TD) perspective. This arises from the “world is green” hypothesis for three trophic level ecosystems, as plants are limited by resources, herbivores are limited by predators, and predators are limited by herbivore abundance (Fretwell, 1977; Hairston, 1989; Hairston et al., 1960; Oksanen, 1983, 1988; Oksanen et al., 1981; Slobodkin et al., 1967). The result is a trophic cascade as predators increase plant abundance by reducing herbivores. An alternative hypothesis proposed is bottom‐up control (“world is brown”) where herbivores do not limit plant abundance and predators do not limit herbivores so that resources limit plant abundance, plant abundance limits herbivores, and herbivore abundance limits predators (Chase, 2000; Hunter & Price, 1992; Leibold, 1989; Matson & Hunter, 1992; Schmitz et al., 2000).

It has been proposed that multiple TD and bottom‐up processes (e.g., compensatory mortality, selective feeding, fear behavior, consumers changing nutrient availability, plant defenses) operate at each trophic level (plants, herbivores, or predators) (Chesson & Kuang, 2008; Feng et al., 2024; Leroux & Loreau, 2015; Lichtenstein et al., 2024; Lynam et al., 2017; Rogers et al., 2020; Rosemond et al., 2001; Rosenblatt & Schmitz, 2016; Shurin, 2012; Wollrab et al., 2012). Therefore, the net effect of these processes at each trophic level determines whether the trophic level will increase or decrease with predation, which implies that there can be greater variety of overall ecosystem control than the dichotomy of either TD or bottom‐up (Leroux & Loreau, 2015; Shurin, 2012). For example, in TD ecosystems, predators decrease herbivores and increase plants, and in bottom‐up ecosystems, predators have no effect on herbivores or plants; however, what if predators decrease herbivores, but do not increase plants, or predators do not decrease herbivores, but increase plants (Hines & Gessner, 2012; Maurya & Priyadarshi, 2021; Schmitz et al., 2000).

A popular approach for identifying trophic possibilities is to experimentally remove or add predators to assess whether herbivore abundance increases or decreases and plant abundance correspondingly decreases or increases to clearly identify the effect of predators (Peckarsky et al., 2008). These experiments often suffer from being short term or spatially restricted (Rogers et al., 2020). In an attempt to overcome temporal and spatial restrictions, multiple studies can be combined to assess whether a particular ecosystem trophic control is observed more frequently. This has been done using vote‐counting (greater number of studies finding a particular trophic control), meta‐analysis (effect sizes in studies on average support a particular trophic control), and correlation (environmental correlates associated with particular trophic controls) (Koricheva & Gurevitch, 2014; Rogers et al., 2020). However, another problem is identifying contextual (variable) explanations of what might produce the alternatives observed in each individual study (Belovsky et al., 2004; Hilborn & Mangel, 1997; Karban & Huntzinger, 2006). Contextual explanations can include seasonal, annual, climate, habitat, food‐type, taxa, and productivity differences (e.g., Boyer et al., 2003; Gratton & Denno, 2003; Meserve et al., 2003; Rogers et al., 2020; Vidal & Murphy, 2018).

Plants, grasshoppers (Orthoptera, Acrididae), and their predators (birds and spiders) may represent a model three trophic‐level terrestrial system for examining trophic dynamics, as they can be manipulated easily and respond rapidly (Belovsky & Joern, 1995). Furthermore, grasshoppers are important herbivores, and may be predator‐limited, given that they are highly sought‐after prey, and have many predators that are larger than them (e.g., birds), making their capture relatively easy. Most studies of these systems are short term (1–2 years) at a single location as is typical of most experimental trophic studies, with some claiming TD and others bottom‐up control (Belovsky & Joern, 1995), but most examine only grasshopper abundance. A few studies suggest that trophic dynamics vary over time and space (e.g., Belovsky & Slade, 1993; Branson, 2005; Joern, 1992; Ritchie, 2000).

To overcome the experimental issues of temporal and spatial restrictions and the need to examine entire system responses, we conducted predator (bird) removal over 34 years (1985–2019, except 1988) in four bunchgrass prairie habitats (NBR: National Bison Range, MT, USA) and measured plant, grasshopper, and spider abundances (NBR monitored bird abundance for 16 of the years). We observed whether plant and grasshopper abundances increased or decreased without birds, which when combined indicate a variety of whole‐system trophic dynamics beyond TD or bottom‐up, that change over time and space, and can be correlated with environmental conditions. The plant and grasshopper overall system responses often were not consistent with either TD or bottom‐up control. Finally, using vote counting, we compared our results over time and space to grasshopper, plant, and predator studies in the literature, as well as literature studies for other systems.

## STUDY SITE

NBR contains one of the largest tracts (~9000 ha) of intermountain bunchgrass prairie remaining in North America. Elevation ranges from 800 to 1400 m. The area has never been farmed and has been protected since 1908 when established as a wildlife refuge for the preservation of bison (*Bison bison*). Prior to this, NBR had been an open‐range cattle ranch for less than 15 years.

NBR climate and vegetation are described elsewhere (Belovsky & Slade, 2020). Unlike many bunchgrass prairies (Daubenmire, 1970), big sagebrush (*Artemisia tridentata*) is absent. Since 1978, annual grass live biomass ranged from 41% to 97%, and aboveground annual net primary production (ANPP) ranged from 100.9 to 316.4 g‐dry/m^2^ (Belovsky & Slade, 2020). Four sites representing different habitats based on dominant vegetation and soil account for >80% of NBR bunchgrass prairie: Site (A) *Poa pratensis* and *Pascopyrum smithii* provide >80% of ANPP, sandy soil; Site (B) *Stipa columbianus* and *P. smithii* provide >70% of ANPP, organic soil; Site (C) *P. smithii* and *Festuca idahoenis* provide >80% of ANPP, rocky/clay soils; Site (D) *P. pratensis*, *Poa compressa*, and *P. smithii* provide >90% of ANPP, clay soils.

Grasshoppers are the dominant herbivores in bunchgrass (Belovsky & Slade, 2024a; Rogers & Uresk, 1974; Sheldon & Rogers, 1978; Shelford, 1963). There are >25 grasshopper species at NBR, all are univoltine, and all but two overwinter as eggs. A late spring (May–June) assemblage of five species is never abundant (≪1 individual/m^2^). A summer assemblage (June–October) is very abundant (≫5 individuals/m^2^) and one species (*Melanoplus sanguinipes*) dominates (50%–90% of biomass). Grasshoppers are more abundant (~5 g/m^2^), but annually more variable, than mammalian herbivores (~2.5 g/m^2^), which leads to 1.25–1.67 times greater plant loss than from mammals (Belovsky & Slade, 2024a). Birds are the major predators of NBR grasshoppers with spiders much less important (Belovsky et al., 1990; Belovsky & Slade, 1993).

## METHODS

### Field measures

Annual vegetation ANPP at each site was measured (Belovsky & Slade, 2020) along with an estimate of hatching grasshoppers (Belovsky & Slade, 2024a). Estimates of NBR passerine bird abundance are available for some years (U.S.F.W.S. surveys).

### Avian exclusion

**FIGURE 1:**
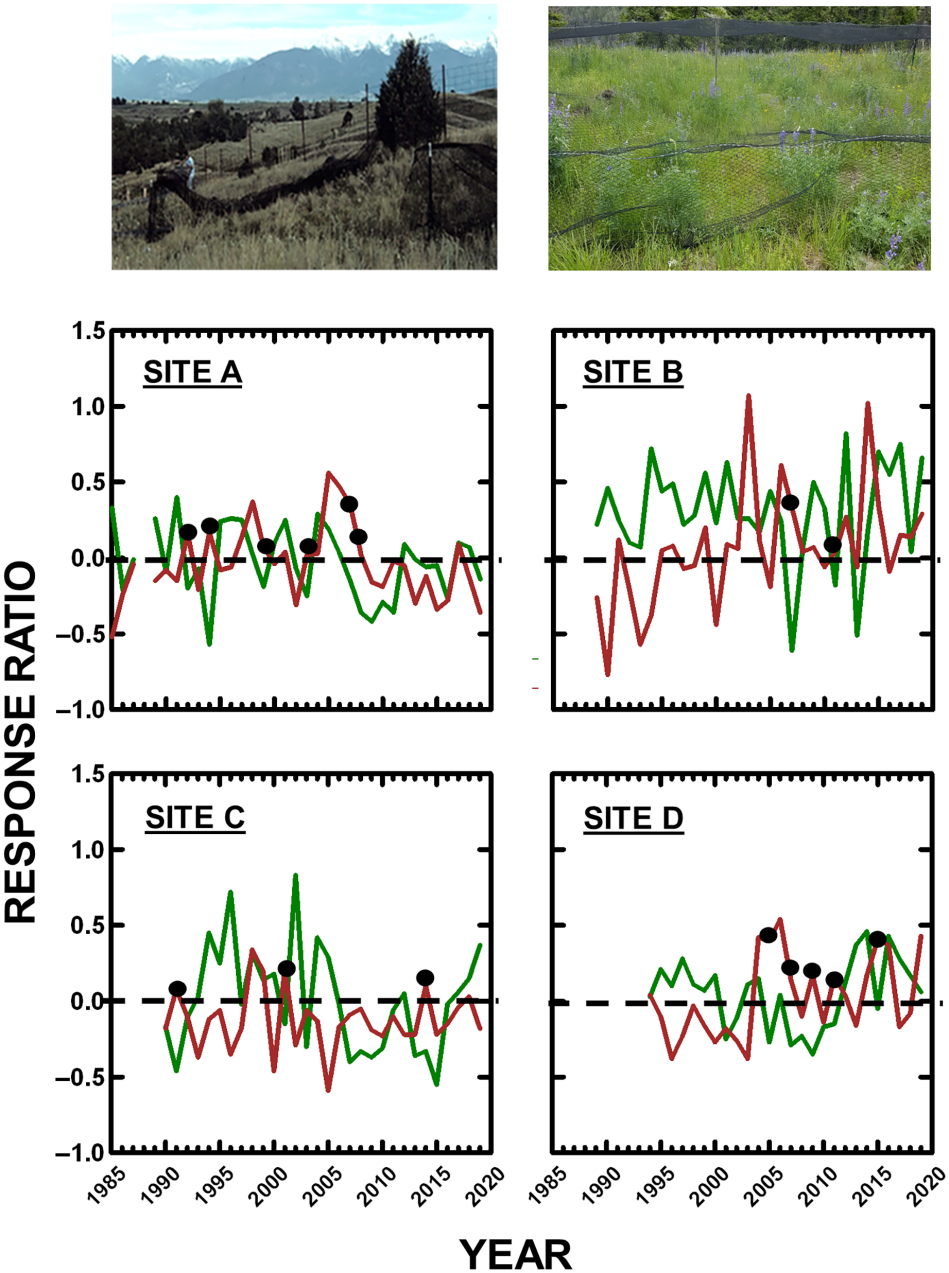
Photos show (left) construction of and (right) completed bird exclosure. The plant biomass (green lines) and total grasshopper abundance (brown lines) response ratios (ln[exclosure/control]) are plotted over time for each site. Datapoints identify when top‐down control of the system is observed (plants decrease and grasshoppers increase). Photo credit: J. B. Slade.

Over 34 years (1985–2019, except 1988), plant, grasshopper, and spider abundances were compared between bird exclosure (photos in Figure 1) and control areas. Experimental design and absence of aberrant effects are provided elsewhere (Belovsky & Slade, 1993; Joern, 1986). In May, we constructed three 100 m^2^ exclosures at each of the sites using nylon mesh (5 cm mesh openings) to exclude birds. Each exclosure was contiguous on one side with a 100‐m^2^ unenclosed area (control). Exclosure–control pairs were randomly assigned at a site.

In late September, insect netting (1.25 m‐high) was placed around each exclosure and control, and the exclosure cover was removed. Insect netting contained escaping grasshoppers or forced them to fly high enough to be observed. Two individuals with insect nets caught grasshoppers in each exclosure and control for three or four 15‐ or 20‐min periods, separated by 10 min to allow remaining grasshoppers to be undisturbed. Grasshopper density was estimated using a “catch‐effort” technique (Southwood & Richard, 1978, p. 230). The sum of all grasshoppers caught in an area prior to each 15–20‐min catch period was the independent variable, and the number caught in the 15–20‐min period was the dependent variable. The three to four pairs of values in a regression provided a population estimate as the *x*‐intercept with a 5%–20% CV.

Grasshoppers were preserved in 70% ethanol for later identification to species (Brooks, 1958; Brusven, 1972; Handford, 1946; Hebard, 1928, 1934; Helfer, 1963; Otte, 1981, 1984; Pfadt, 2002; Scoggan & Brusven, 1972; Scott, 2010), life stage, sex, and adult body size (large: ≥500 mg live; medium: <500–>250 mg live; small: ≤250 mg live). Species richness (number of species) and diversity (Shannon index = −Σ*p*_*i*_ × ln*p*_*i*_, where *p*_*i*_ is species *i*'s relative abundance) were computed. We also counted all spiders observed with an abdominal diameter >5 mm, as an index of spider abundance (the primary invertebrate predator) (Belovsky et al., 1990).

After censusing grasshoppers, vegetation (green) inside each exclosure and control area was clipped in five 0.1 m^2^ plots that were located randomly (one per quadrant and one in the center). Clipped vegetation was separated between grasses and forbs, as they differ in nutritional value and grasshopper species differ in selective feeding on them (Belovsky, 1986a). Plant samples were dried, weighed, and chemically digested in HCl and pepsin (an index of digestibility to grasshoppers: Belovsky & Slade, 1995). An index of food abundance (edible biomass) for grasshoppers in each area is the sum of grass biomass times its solubility and forb biomass times its solubility (Belovsky & Slade, 1995). Finally, to estimate plant cover, an observer walked along the diagonals within each exclosure and control area, and with each step, paused and recorded whether the toe of the shoe touched vegetation (regardless of being green or brown) or bare soil until 25 toe‐points were recorded (Daubenmire, 1947).

### Data analysis

The two most important trophic measures for the bird exclusion experiments are plant biomass and total grasshopper abundance. These values for exclosures minus controls are always greater than zero so that effect size can be measured as ln response ratios (exclosure/control). More fine‐grained measures (large and small grasshoppers, and spiders) sometimes are zero (exclosure or control), which means that ln response ratios cannot be used (zero values produce undefined or ∞ values), so differences (exclosure − control) were employed. In both cases, negative effect sizes indicate that values for controls are greater than values for exclosures. Signs of effect sizes for each year and site, across all years at a site, and across all sites and years are compared with expected TD responses (plant value <0, grasshopper value >0) using *t* tests (≠0, one‐sided *p* < 0.05). Effect sizes across all sites and years were examined using general linear models (GLM) for site and year effects, and post hoc pairwise site differences were examined. Finally, spatial concordance in plant biomass and total grasshopper abundance trophic patterns were examined over time using correlation analysis.

Each site/year's plant biomass and total grasshopper abundance effect size signs can be used to designate TD (plant value <0, grasshopper value >0) or bottom‐up (plant value ≥0, grasshopper value ≤0) control. These site/year designations for plants and grasshoppers can be related to various environmental factors (e.g., ANPP, plant biomass, plant edible‐biomass, plant cover, grasshopper hatchling abundance, spider abundance) using a combination of decision tree and discriminant analyses (Feldesman, 2002; Guler et al., 2001; Suner et al., 2012; Todeschini & Marengo, 1992; Yildiz & Alpaydin, 2005). Successful prediction of trophic responses for plant biomass and total grasshopper abundance can be addressed using χ^2^ tests (correct number predicted vs. incorrect number).

Whether overall ecosystem trophic structure was TD can be assessed using (1) only the sign of plant biomass effect size (TD: <0), (2) only the sign of total grasshopper effect size (TD: >0), and (3) both the sign of plant biomass and total grasshopper effect sizes. These predictions provide counts (votes) for the frequency that system‐wide trophic responses are TD. Finally, studies summarizing trophic structure reported in the literature using the different methods can be compared with the results obtained in our experiments, where the frequency of trophic structures can be compared using χ^2^ contingency tables (trophic structure counts for our study vs. literature studies).

Data were transformed if necessary to achieve normality; all proportions were arcsine square root transformed. Statistics were performed using SYSTAT 13.

## RESULTS

Site/year measures are available from Dryad (https://doi.org/10.5061/dryad.ghx3ffbvh). The computed effect sizes are presented in Appendix S1: Table S1. The plant and total grasshopper effect sizes over time for each site are plotted together in Figure 1.

### Vegetation biomass

Vegetation biomass, based on effect sizes across all sites (Figure 1), on average, does not exhibit a TD response (ln[exclosure plant biomass, no birds/control plant biomass, with birds] < 0: 0.08 ± 0.03 [mean ± SE], *p* < 0.005, df = 360), which is not surprising as other experiments at these sites demonstrate that water and nitrogen limit plant ANPP, a bottom‐up response (Belovsky & Slade, 2000, 2002, 2018, 2020; Schmitz, 1993; Volenec & Belovsky, 2018). Nonetheless, TD responses are periodically exhibited at all sites (38.8%, 47/121 cases), but less often than not (χ^2^ = 6.02, df = 1, *p* < 0.01), and there is no difference if only statistically significant effect sizes are examined (TD = 37.5%, 15/40 cases, χ^2^ = 0.02, df = 1, *p* < 0.88). TD responses on average decrease plant biomass in exclosures by 19.8% ± 2.0%, and the more frequently observed increase in plant biomass averaged 35.9% ± 3.7%. Effect sizes differ among years (categorical: *F* = 1.33, *p* = 0.05, df = 33, 324) and sites (*F* = 8.45, *p* = 0.00001, df = 3, 324), with Sites A, C, and D less likely to exhibit TD responses than Site B. Plant biomass responses among sites within a year tend to agree (possible range of 2–4 cases/year) (1994–2019: mean of 3.2 agree/year: *t* = 8.1, df = 25, *p* < 0.000001). Finally, plant communities based on relative grass abundance ([exclosure − control]/control) did not differ whether a TD response was (2.9% ± 4.2%) or was not observed (−0.9% ± 3.1%).

### Grasshopper abundance

Grasshopper total abundance, based on effect sizes across all sites (Figure 1), on average, does not exhibit a TD response (ln[exclosure grasshopper abundance, no birds/control grasshopper abundance, with birds] > 0: −0.02 ± 0.02, *p* < 0.30, df = 360). However, TD responses are periodically exhibited at all sites (41.3%, 50/121 cases), but less often than not (χ^2^ = 3.6, df = 1, *p* < 0.05), and there is no difference if only statistically significant effect sizes are examined (TD = 37.5%, 12/45 cases, χ^2^ = 3.01, df = 1, *p* < 0.09). TD responses (increased grasshopper abundance without birds) led to an increase of 29.9% ± 5.9% on average, while the more frequently observed decrease in grasshopper abundances without birds was 16.9% ± 1.3% on average. Effect sizes differ among years (categorical: *F* = 2.04, *p* = 0.0005, df = 33, 324) and sites (*F* = 3.91, *p* = 0.005, df = 3, 324), with Sites A, B, and D more likely to exhibit TD responses than Site C. Grasshopper total abundance responses among sites within a year tend to agree (possible range of 2–4 cases) (1994–2019: mean of 2.6 agree/year: *t* = 4.8, df = 25, *p* < 0.00006). Finally, whether a TD response was or was not observed, grasshopper species richness without birds (respectively increased, 9.7% ± 4.5% and 7.3% ± 6.6%) and diversity without birds (respectively increased, 2.6% ± 3.1% and 8.8% ± 5.1%) did not differ.

Large‐bodied adults (≥500 mg), averaging only 4.5% ± 0.5% of grasshoppers in exclosures, more frequently exhibit TD responses at all sites (64.5%, 78/121 cases) than not (χ^2^ = 10.12, df = 1, *p* < 0.001). Effect sizes for large adults differ among years (categorical: *F* = 5.71, *p* = 0.0004, df = 33, 324), but not among sites (*F* = 0.73, *p* = 0.43, df = 3, 324). Small‐bodied adults (≤250 mg), averaging only 11.1% ± 0.9% of grasshoppers in exclosures, less frequently exhibit TD at all sites (35.5%, 43/121) than not (χ^2^ = 10.12, df = 1, *p* < 0.001). Effect sizes for small adults differ among years (*F* = 1.69, *p* = 0.006, df = 33, 324), but not among sites (*F* = 0.87, *p* = 0.23, df = 3, 324). These size‐based differences in the frequency of TD control are expected as birds preferentially prey upon large‐bodied grasshoppers at NBR (Belovsky et al., 1990; Belovsky & Slade, 2010). Small adults tend to decrease in abundance when large adults increase in abundance (81.8% of time, 45/55 cases) (χ^2^ = 22.27, df = 1, *p* < 0.000002), which is consistent with the known competitive superiority of larger‐bodied grasshoppers at these sites (Belovsky, 1986b, 1997; Belovsky et al., 2011; Belovsky & Slade, 1995; Chase, 1996a; Chase & Belovsky, 1994).

### Predators

Spider abundances based on effect sizes, on average, exhibit a strong TD response (ln[exclosure spider abundance, no birds/control spider abundance, with birds] > 0: 2.37 ± 0.32, *p* < 0.000001, df = 360). TD responses are much more frequently observed (76.9%, 93/121 cases) than not (χ^2^ = 34.92, df = 1, *p* < 0.000001) with birds reducing spiders by 73.6% ± 8.2%. Effect sizes for spiders differ among years (*F* = 2.94, *p* = 0.000001, df = 33, 324), and among sites (*F* = 36.71, *p* = 0.000001, df = 3, 324), with Sites A, B, and C exhibiting weaker TD responses than D. Spider abundance responses among sites within a year tend to agree (possible range of 2–4 cases/year) (1994–2019: mean of 3.3 agree/year: *t* = 8.1, df = 25, *p* < 0.000001). Finally, spider abundance annually varies more (CV = 91%–138% by site) than bird abundance (CV = 22%), which suggests that intraguild predation by birds on spiders may explain why spiders are not important grasshopper predators at NBR (Belovsky et al., 1990; Belovsky & Slade, 1993; Chase, 1996b; Schmitz, 1993).

### System traits

System traits driving the two most important trophic responses, plant biomass and total grasshopper abundance with and without birds, must have varied among years and sites (Figure 1), but this is not due to variation in predator abundance (spider or bird). For example, whether grasshoppers exhibit TD responses or not is not related to predator abundance (spiders: df = 119, *t* = 0.85, *p* < 0.38; birds: df = 60, *t* = −1.12, *p* < 0.27). Predator abundance increases with abundance of grasshoppers in exclosures and controls, which suggests that predators are limited by their prey availability, but this is not statistically significant (spiders—GLM with site as a categorical variable: df = 1116, *p* < 0.39 and 0.59; birds—regression: *N* = 62, *p* < 0.33 and 0.35). These results suggest that predator abundance, in and of itself, is not driving the bird exclusion results, but other factors that impact predation intensity may be important.

**FIGURE 2:**
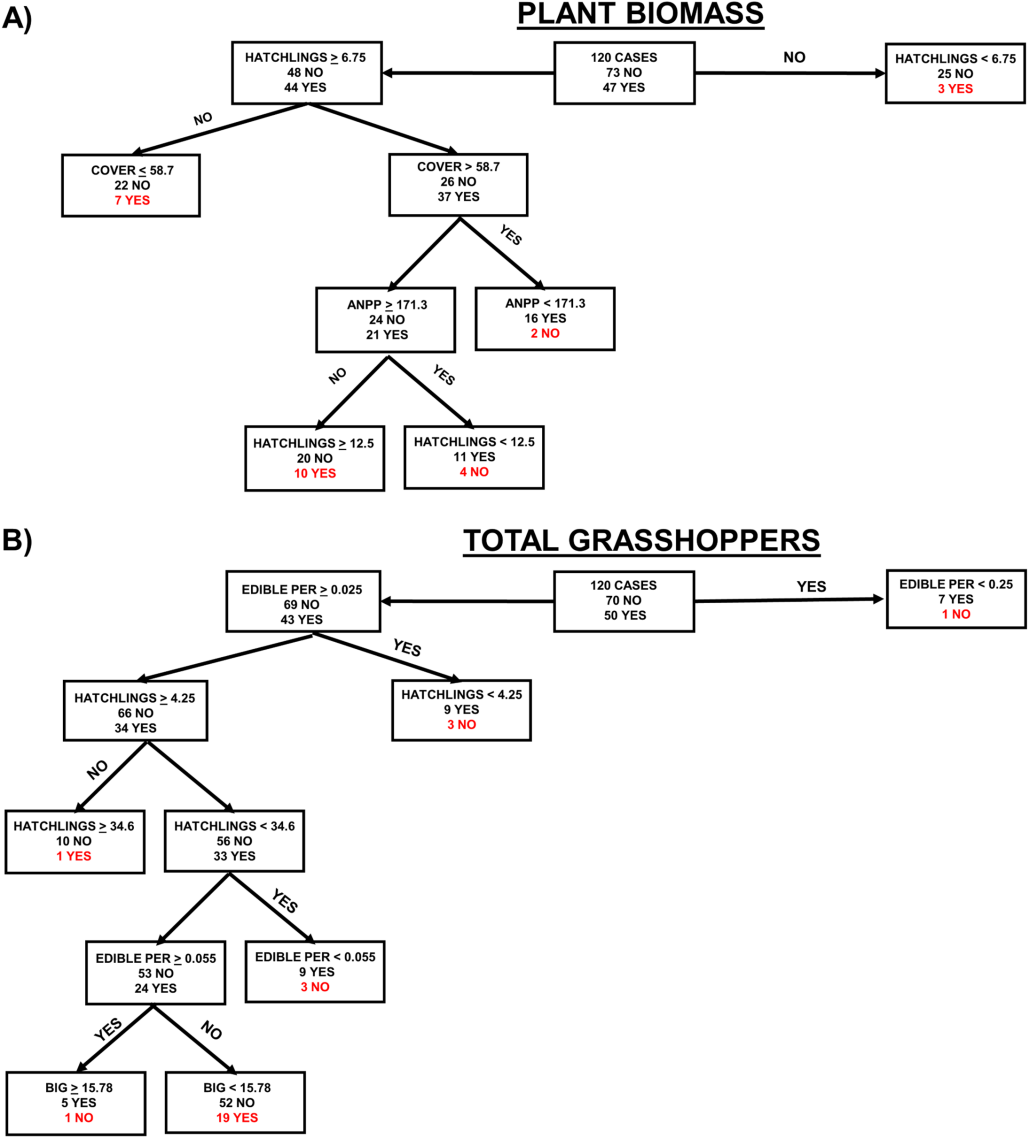
Responses of plants and grasshoppers in exclosures and controls (120 cases) were analyzed using decision tree/discriminant analysis and decision nodes (grasshopper hatchling abundance, plant cover, annual net primary production [ANPP], edible plant biomass per grasshopper hatchling, proportion large‐bodied grasshoppers [BIG]) are indicated. (A) Plants decrease (Yes, expected with top‐down control) or increase (No) with bird exclusion. (B) Total grasshoppers increase (Yes, expected with top‐down control) or decrease (No) with bird exclusion. Incorrect predictions are in red.

The roles played by system traits that can impact predation intensity (e.g., ANPP, edible‐biomass and cover, grasshopper hatchling abundance, percent large grasshopper abundance) were examined using decision tree/discriminant analysis (Feldesman, 2002; Guler et al., 2001; Suner et al., 2012; Todeschini & Marengo, 1992; Yildiz & Alpaydin, 2005). TD responses exhibited by plant biomass tend to occur with lower ANPP, higher grasshopper hatchling abundance, and more cover (78.3% correct, χ^2^ = 38.5, df = 1, *p* < 0.000001; Figure 2A). This contextual response is expected, as grasshoppers are more likely to reduce plant biomass when there is less food for them, more grasshoppers to consume it, and more cover for the grasshoppers to hide from predators, so they can feed more. TD responses exhibited by total grasshoppers tend to occur when there is a lower biomass of edible plants/grasshopper hatchling abundance, when there is a lower grasshopper hatchling abundance, and when large‐bodied grasshoppers make up a greater proportion of the grasshoppers (76.7% correct, χ^2^ = 34.1, df = 1, *p* < 0.000001; Figure 2B). This contextual response is expected, as birds are more likely to reduce grasshopper abundance when there are fewer grasshoppers to begin with, when grasshoppers need to spend more time searching for food (less food per grasshopper), and there are more large‐bodied grasshoppers (preferred by birds). These relationships are supported by predation experiments using tethered NBR grasshoppers (Belovsky et al., 1990, 2011; Belovsky & Slade, 1993, 2010).

### System‐wide trophic response

System‐wide trophic response often is assessed solely on the basis of either plant or herbivore responses. The mean effect size for plant biomass or total grasshopper abundance alone does not indicate a system‐wide TD response (respectively, 0.08 ± 0.03 and −0.02 ± 0.02). However, site/year variation is great and cannot be ignored by averaging, as a TD response of 38.8% is observed with plant biomass, and 41.3% with total grasshopper abundance (Figure 1).

Even more critical, system‐wide trophic response depends on plant biomass and total grasshopper responses combined, not either in isolation. When both plant and grasshopper responses are TD for the same site and year (Figure 1; Appendix S1: Table S2), then TD (plants decrease and grasshoppers increase in exclosures) control is attributed in only 13.2% (16/121) of sites/years. In 33.1% of cases (40/121), plants increased and grasshoppers decreased in exclosures (I–D); in 28.1% of cases (34/121), plants increased and grasshoppers increased in exclosures (I–I); and in 25.6% of cases (31/121), plants decreased and grasshoppers decreased in exclosures (D–D). Using only cases where site/year plant and grasshopper effect sizes are both statistically significant, the same pattern is observed (χ^2^ = 3.86, df = 2, *p* < 0.15; Appendix S1: Table S2). Finally, if plant and grasshopper trophic responses over all sites/years are considered to be independent probabilities, then the expected four combinations of trophic responses (TD, I–D, I–I, D–D) over all sites/years are the product of plant and grasshopper probabilities, which are similar to the observed values (Appendix S1: Table S2). Therefore, more system‐wide trophic possibilities exist than TD and bottom‐up, and these cannot be identified by examining only plant or only grasshopper responses. Furthermore, the spatial and temporal variation in system‐wide responses observed here is obscured if overall mean effect sizes are examined; rather counts of plant and grasshopper combined responses over time and space must be employed to identify this variability.

### Long‐term changing patterns

**FIGURE 3:**
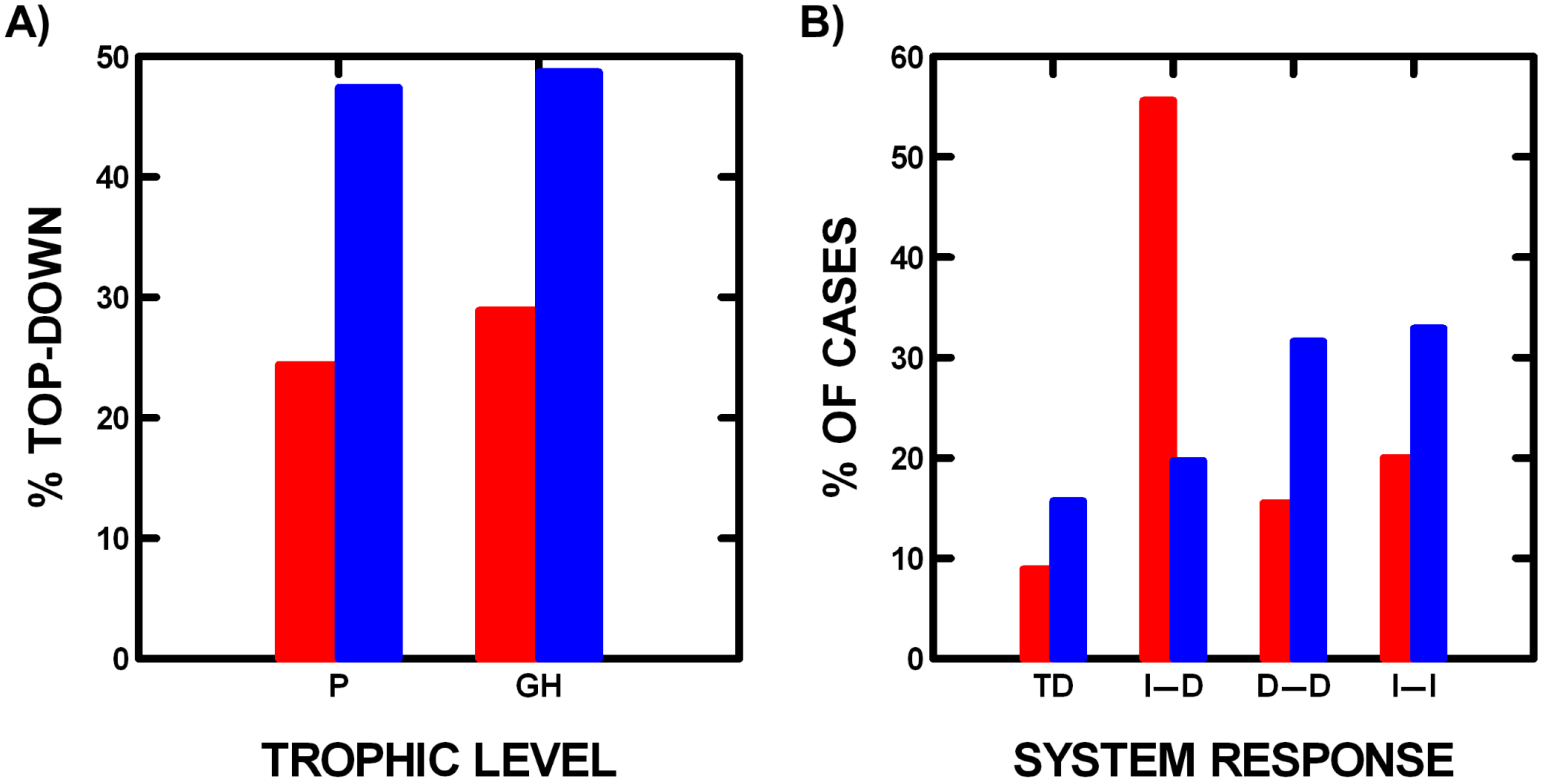
Comparison of the periods 1985–2000 (red bars) and 2001–2019 (blue bars) is presented: (A) plant biomass (P) and total grasshopper abundance (GH) trophic responses (percentage of cases that are top‐down = TD, i.e., where plants decrease or grasshoppers increase with bird exclusion), and (B) combined plant and grasshopper responses (respectively, first value–second value, where D = decrease and I = increase) provide the system‐wide trophic response (percentage of cases when TD = D–I, I–D, D–D, and I–I).

Long‐term changing patterns in trophic dynamics emerged in our study. Plant and grasshopper TD responses have increased since 2000 (respectively, χ^2^ = 6.3, df = 1, *p* = 0.01; χ^2^ = 4.6, df = 1, *p* = 0.03; Figure 3A). This is expected given that climate change over 40 years (1978–2019) decreased plant edible biomass (*p* = 0.0005, Belovsky & Slade, 2020), and as shown above, TD control is more likely when grasshoppers have less food. Finally, changes in plant and grasshopper trophic responses led to system‐wide changes (Figure 3B) with I–D responses declining (χ^2^ = 16.4, df = 1, *p* = 0.00005) and I–I and D–D responses increasing (χ^2^ = 9.5, df = 1, *p* = 0.002).

### Comparison with other trophic studies

Some examples of trophic studies in the literature were compared with our results (Appendix S1: Table S2) for the frequency of TD control of plants or herbivores, and system‐wide dynamics when studies simultaneously measured plants and herbivores or probabilistic projections (see above) when both were not simultaneously studied. First, we reviewed similar grassland, meadow, desert plant → grasshopper → predator experiments, and found that summary values were similar to our results (Appendix S1: Table S2: I vs. II). Second, we summarized data from a classic review of TD observations for plants and herbivores based on counts (Sih et al., 1985) (Appendix S1: Table S2: III), and several meta‐analysis studies (mean effect sizes), where data from individual reported experiments were converted to counts (Appendix S1: Table S2: I vs. IV). We found that our literature survey of observed plant, herbivore, and system‐wide trophic responses or projected system‐wide trophic responses, when combined, produces similar proportions of the different trophic responses as observed in our field study (Appendix S1: Table S2: I vs. V).

This comparison supports our conclusion that system‐wide trophic dynamics cannot be assessed using either plant or herbivore responses alone, as more than system‐wide TD or bottom‐up responses emerge. While the summarized studies are often short‐term and spatially restricted, as criticized by Rogers et al. (2020), they still provide similar results to our long‐term study in four habitats, suggesting that they may “capture” the temporal/spatial variability revealed in our study when combined, not necessarily system differences. Finally, the similar results suggest that the plant–grasshopper–bird system may be a model system for studying trophic dynamics.

## DISCUSSION

Our long‐term (26–34 years) experimental study at multiple sites (four habitats) examines whether plant biomass and total grasshopper abundance exhibit TD (respectively, 38.8% and 41.3% of 121 sites/years) control by birds. In addition, large‐bodied grasshoppers and spiders frequently exhibit TD control (respectively 64.5% and 76.9% of cases), while small‐bodied grasshoppers infrequently exhibit TD control (35.5% of cases). Considerable annual and habitat variation is observed so that when plant and total grasshopper abundance changes without birds are combined to examine system‐wide control, TD control (plants decrease and grasshoppers increase with bird exclusion) only occurred in 13.2% of cases. In most cases, combined plant and grasshopper responses differed from TD expectation (I–D: plants increase and grasshoppers decrease with bird exclusion in 33.1% of cases; I–I: both plants and birds increase with bird exclusion in 28.1% of cases; D–D: both plants and grasshoppers decrease with bird exclusion in 25.6% of cases). Therefore, these systems temporally and spatially exhibit a much greater variety of responses than the often‐considered dichotomy of TD versus bottom‐up control. Finally, over time (1985–2019), the frequency of TD control of plants and grasshoppers has increased.

Our observed system‐wide responses are consistent with a review of trophic studies in the literature. Consequently, the generality of TD, “the world is green” (Fretwell, 1977; Hairston, 1989; Hairston et al., 1960; Hairston & Hairston, 1993; Oksanen, 1983, 1988; Oksanen et al., 1981; Oksanen & Oksanen, 2000; Slobodkin et al., 1967), versus bottom‐up, “the world is brown” (Chase, 2000; Hunter & Price, 1992; Leibold, 1989; Matson & Hunter, 1992; Schmitz et al., 2000), views of terrestrial system‐wide control must be questioned, as a greater range of possibilities exist. Therefore, the issue arises as to what creates the variety of trophic responses.

A possibility is that this is a four trophic‐level system (plants → grasshoppers → spiders → birds), which is common in aquatic systems (Carpenter et al., 1985, 1987; Fretwell, 1977, 1987; Hairston & Hairston, 1993), rather than a three trophic‐level system (plants → grasshoppers → birds). We discount this, given spider experiments at these sites. First, spiders do not limit grasshoppers in cages containing grasshoppers and spiders alone at observed field densities (Belovsky et al., 1990; Belovsky & Slade, 1993; Chase, 1996b; Schmitz, 1993). Second, we found that the field densities of spiders are dramatically reduced by birds (69.2%) so that intraguild predation by birds on spiders converts a potential four trophic‐level system to a three trophic‐level system.

Next, a question arises as to why we never observe system‐wide bottom‐up responses (0, 0). Based on short‐ and long‐term experiments and monitoring, we know that plant ANPP increases as water and nitrogen availability increase, and the amount of these resources differs among sites and years, a bottom‐up pattern (Belovsky & Slade, 2000, 2002, 2018, 2020; Schmitz, 1993; Volenec & Belovsky, 2018). We also know that grasshopper and other herbivore abundances are increased at these sites as plant ANPP increases, a bottom‐up pattern (Belovsky & Slade, 2024a). This suggests that TD system‐wide responses should not be common, which is what we observe. However, multiple TD and bottom‐up modifying processes can operate at each trophic level in a complex ecosystem and their net effects can produce a variety of system‐wide trophic controls, as we observe (Chesson & Kuang, 2008; Feng et al., 2024; Leroux & Loreau, 2015; Lichtenstein et al., 2024; Lynam et al., 2017; Rogers et al., 2020; Rosemond et al., 2001; Rosenblatt & Schmitz, 2016; Shurin, 2012; Wollrab et al., 2012).

A number of potential processes that can influence the variety of trophic responses (Schmitz, 2010) have been experimentally identified by us at our sites. There are three processes (1 through 3) that are ubiquitous across sites, and two processes (4 and 5) that differ among sites:

1. Grasshoppers at our sites often consume all edible‐plant biomass regardless of their abundance (Belovsky & Slade, 1995; Schmitz, 1993), a potentially important trophic dynamic, because decreases or increases in herbivore numbers would have no effect on plant abundance (Leibold, 1989; Schmitz, 1992).
2. Grasshopper abundance at our sites often increases with predation (58.7% of cases: χ^2^ = 3.6, df = 1, *p* = 0.05), because per capita food is low and predation reduces food competition (Belovsky & Slade, 1993; Belovsky et al., 2011; Kistner & Belovsky, 2014). This increases survival, because starvation affects all individuals equally and is greater than predatory mortality, which only affects specific individuals (Łomnicki, 1988). We also know that bird predation tends to preferentially remove individual grasshoppers that have not fed well (Belovsky & Slade, 2010).
3. Grasshoppers at our sites change their activity patterns and time spent feeding with predators (fear response), which can modify their vulnerability to predators and consumption of plants, especially when per capita food is already low (Belovsky et al., 2011). This has been suggested to be an important dynamic in trophic systems (Schmitz, 2010; Schmitz et al., 1997).
4. Grasshoppers at our sites have a very strong effect on nitrogen availability for plants that varies with plant species composition of the community due to plant species differing in decomposition rate and grasshopper selective feeding on them (Belovsky & Slade, 2000, 2002, 2018, 2019). At Sites B and D, grasshoppers tend to increase nitrogen availability and here plant biomass tends to increase (I–D and I–I) in 78.9% of cases, while at Sites A and C, grasshoppers tend to decrease nitrogen availability, and here, plant biomass tends to increase in only 45.3% of cases (χ^2^ = 14.36, df = 1, *p* = 0.0002). Furthermore, sites where grasshoppers increase nitrogen availability have response magnitudes more than double magnitudes at sites where grasshoppers decrease nitrogen availability (plant: *t* = 3.09, *N* = 63, *p* < 0.003; grasshopper: *t* = 2.28, *N* = 63, *p* < 0.03).
5. Grasshopper abundance tends to be reduced when large‐bodied grasshoppers are abundant, because large‐bodied grasshoppers are competitively superior to smaller grasshoppers; however, birds preferentially kill large‐bodied grasshoppers reducing their abundance and competitively releasing smaller grasshoppers, which should increase total grasshopper abundance (Belovsky, 1986b, 1997; Belovsky et al., 1990, 2011; Belovsky & Slade, 1993, 1995, 2010; Chase, 1996a; Chase & Belovsky, 1994). This is the classic competitive “size‐efficiency” hypothesis (Brooks & Dodson, 1965; Chase et al., 2002; Hall et al., 1976). Therefore, bird exclusion should increase large‐bodied grasshopper abundance, and reduce total grasshopper abundance, as observed. This should decrease nitrogen availability and plant biomass (D–D) where grasshoppers positively affect nitrogen availability (Sites B and D), and increase nitrogen availability and plant biomass (I–D) where grasshoppers decrease nitrogen availability (Sites A and C), as observed (χ^2^ = 6.04, df = 1, *p* = 0.01).

All of the above processes (1–5) interact to produce the variety of system‐wide trophic responses: TD (D–I), I–D, I–I, and D–D, and failure to observe bottom‐up responses (0–0). Critical for producing this variety of responses is the effect of grasshoppers on nitrogen availability for plants (process 4), because at sites where grasshoppers increase nitrogen, nitrogen availability increases up to 22% and ANPP increases up to 33% above sites where grasshoppers decrease nitrogen availability (Belovsky & Slade, 2018, 2019). Furthermore, comparing 20 NBR sites, we found the frequency of when grasshoppers increase versus decrease *N* availability is comparable (Belovsky & Slade, 2018).

Beyond categorizing the variety of system‐wide responses and the processes influencing them, response magnitudes (percentage change) can also be examined. First, when plant biomass declines with bird exclusion, plant biomass and total grasshoppers respond comparably: TD (D–I: 23.1% ± 3.6% vs. 18.6% ± 3.6%: *t* = 0.88, df = 30, *p* = 0.39), and D–D (18.1% ± 2.4% vs. 15.3% ± 1.4%: *t* = 1.02, *N* = 60, *p* = 0.31). When plant biomass increases with bird exclusion, plant biomass responds much more than when it declines. Meanwhile, when grasshoppers increase, the grasshopper response is comparable to the plant biomass response (I–I: 39.6% ± 6.2% vs. 35.2% ± 8.4%: *t* = 0.42, *N* = 66, *p* = 0.68), but when grasshoppers decline, the response is much less than the plant response (I–D: 32.8% ± 4.2% vs. 18.2% ± 2.0%: *t* = 3.12, df = 78, *p* = 0.003), and comparable to the grasshopper response with D–I and D–D (see above). Therefore, most responses (D–I, D–D, and I–I) are constant down the food chain, but increase down the food chain for I–D. This differs from claims that responses decrease down the food‐chain (Schmitz et al., 2000).

## CONCLUSION

Our observation of a much greater variety of system‐wide trophic responses than TD and bottom‐up and the low frequency of TD responses (13.2%) is supported by similar results observed with our summary of literature reports. This variety of responses should be expected given the diversity of processes observed to operate in complex ecosystems (Chesson & Kuang, 2008; Feng et al., 2024; Leroux & Loreau, 2015; Lichtenstein et al., 2024; Lynam et al., 2017; Rogers et al., 2020; Rosemond et al., 2001; Rosenblatt & Schmitz, 2016; Shurin, 2012; Wollrab et al., 2012). Furthermore, we find system‐wide trophic dynamics cannot be assessed solely on either plant or herbivore responses (Belovsky & Joern, 1995; Bridgeland et al., 2010; Schmitz, 2010; Schmitz et al., 2000, 2006). Finally, we observe considerable spatial and temporal variability in these responses. Therefore, the “world is green” (TD) or “brown” (bottom‐up) are not simple alternatives (Chase, 2000; Chase et al., 2002; Fretwell, 1977; Hairston, 1989; Hairston et al., 1960; Hunter & Price, 1992; Leibold, 1989; Matson & Hunter, 1992; Oksanen, 1983, 1988; Oksanen et al., 1981, 2001; Pimm, 1982, 1991; Polis et al., 2000; Polis & Winemiller, 1996; Power, 2000; Schmitz et al., 2000, 2008; Schoener, 1989; Slobodkin et al., 1967), as often found in ecology (Belovsky et al., 2004; Hilborn & Mangel, 1997; Karban & Huntzinger, 2006).

System‐wide trophic responses can be difficult to identify using manipulative or “natural” (few similar sites) experiments for some systems (e.g., large herbivores and predators). However, system traits such as plant, herbivore, and predator abundances when combined with various processes may help to identify system‐wide responses. Some of the processes proposed include (1) feeding behavior (Beckerman, 2002; Elmhagen et al., 2010; Laws et al., 2009; Logan et al., 2006; Preisser et al., 2009; Rothley et al., 1997), (2) fear of predators (McArthur et al., 2014; Schmitz et al., 1997, 2004; Trussell et al., 2008; Wirsing et al., 2021), (3) herbivore consumption of all edible plants regardless of herbivore density (Leibold, 1989; Schmitz, 1992), (4) multiple types of predators (Pitt, 1999; Schmitz, 2010; Schmitz & Sokol‐Hessner, 2002), (5) keystone predation reducing competitively superior herbivores (Chase et al., 2002; Paine, 1966; Power & Mills, 1995), (6) predation reducing more severe herbivore mortality due to food shortages (Errington, 1946a, 1946b; Łomnicki, 1988), and (7) consumers affecting nutrients for plant growth (Rosenblatt & Schmitz, 2016; Schmitz, 2010). We observed all of these processes to operate with the latter three most important. Finally, these processes explain why ANPP, plant cover, edible biomass per grasshopper hatching, total grasshopper abundance, and the abundance of large grasshoppers explain plant and grasshopper responses using decision tree/discriminant analysis.

Finally, if there exists a greater variety of system‐wide trophic responses than TD or bottom‐up, but these are the only two responses considered in developing management plans by applied ecologists, unanticipated results may be expected (Allen et al., 2014; Branson et al., 2006; Cornwall, 2024; Johnson, 2010; Kirk et al., 1996; Miller et al., 2001; Prugh et al., 2009; Ritchie et al., 2012; Ritchie & Johnson, 2009; Schmitz, 2005; Sergio et al., 2008; Stier et al., 2016; Tahir et al., 2017).

## CONFLICT OF INTEREST STATEMENT

The authors declare no conflicts of interest.

## Supporting information


Appendix S1.

## Data Availability

Data for site/year measures (Belovsky & Slade, 2024b) are available from Dryad: https://doi.org/10.5061/dryad.ghx3ffbvh.
