# Supplementary material for: How often are ecosystems top‐down controlled? Experiments in grassland, grasshopper, and bird systems over time and space
Source: Ecosphere. 2024 Nov 21;15(11):e70066. doi: 10.1002/ecs2.70066 (PMC11583287; doi:10.1002/ecs2.70066)
Supplement: Supplementary file 1 — Appendix S1. [file ECS2-15-e70066-s001.pdf]

Authors: Gary E. Belovsky and Jennifer B. Slade
Manuscript title: HOW OFTEN ARE ECOSYSTEMS TOP-DOWN CONTROLLED? Experiments in grassland, grasshopper and bird systems over time and space
Journal name: ECOSPHERE

APPENDIX S1: TABLE S1. Plant biomass[a], grasshopper abundance (total[a], large-bodied[b], small-bodied[b]), and spider abundance[b] responses (decline or increase) with bird exclusion for all 4 sites. Superscripts refer to the measures: a) ln response ratios (exclosure/control) or b) deviation (exclosure - control). These effect sizes were compared statistically with top-down expectations (plants < 0, grasshoppers > 0: one-sample t-test, * $p_{one\text{-}sided} < 0.05$).

Continuation of APPENDIX S1: TABLE S1.

	PLANTS[a]				GRASSHOPPERS OVERALL[a]				LARGE[b]			
SITE	A	B	C	D	A	B	C	D	A	B	C	D
YEAR												
1985	0.33	……	……	……	-0.52*	……	……	……	1.46	……	……	……
1986	-0.21	……	……	……	-0.24*	……	……	……	0.00	……	……	……
1987	-0.01	……	……	……	-0.04	……	……	……	1.45	……	……	……
1988	……	……	……	……	……	……	……	……	……	……	……	……
1989	0.26[c]	0.22	……	……	-0.15*	-0.26*	……	……	0.73	0.63*	……	……
1990	-0.09	0.46	-0.17	……	-0.08	-0.77*	-0.18	……	0.53*	1.42	0.88	……
1991	0.40	0.25	-0.46*	……	-0.15*	0.12	0.08	……	0.68	8.15*	-0.04	……
1992	-0.20	0.10	-0.11	……	0.16	-0.21*	-0.10	……	3.48*	0.05	3.33*	……
1993	-0.07	0.07	0.03	……	-0.21*	-0.57*	-0.37	……	1.40	2.95	2.3	……
1994	-0.57[b]	0.72	0.45*	0.03	0.17	-0.38*	-0.12	0.04	1.11*	0.36	-0.97*	1.97*
1995	0.24[b]	0.44	0.25	0.21	-0.08	0.05	-0.06	-0.10	2.10	1.70	-0.31	0.24
1996	0.26	0.49*	0.72	0.10	-0.06	0.08	-0.35*	-0.38*	0.02	6.13	-2.95	-4.31
1997	0.25	0.22	-0.02	0.28*	0.14*	-0.07	-0.18	-0.23*	-1.11*	3.67	3.5	-2.17
1998	0.02	0.28	0.32*	0.11*	0.37*	-0.05	0.34	-0.03	1.87*	1.74	5.57	-2.45
1999	-0.19	0.56	0.14*	0.07	0.07	0.20	0.20	-0.16*	1.48	-1.85	2.65	-1.86
2000	0.10	0.23	0.18	0.17*	-0.04	-0.44*	-0.46	-0.27*	0.74*	0.67	-0.99*	0.04
2001	0.25	0.63*	-0.15	-0.25*	0.04	0.09	0.21	-0.18	1.48*	0.34	-5.80*	5.06
2002	-0.07	0.26*	0.83*	-0.11	-0.31*	0.06	-0.29*	-0.26*	1.10*	1.76	-3.27*	-1.10
2003	-0.25	0.26*	-0.30[c]	0.11	0.02	1.07*	-0.06	-0.38*	0.37	4.69	2.05*	-0.34
2004	0.29[b]	0.17	0.42	0.15	0.03	0.11	-0.13*	0.42*	0.38	0.82*	0.65	0.85
2005	0.19	0.44*	0.29	-0.27*	0.56*	-0.19	-0.59*	0.40	0.49*	4.95	-0.68*	0.33
2006	0.03	0.24	-0.02	0.04	0.47	0.61*	-0.17*	0.54*	1.46	10.78	0.33	0.33
2007	-0.15	-0.61*	-0.40*	-0.29	0.35*	0.33	-0.09	0.14	0.00	0.33	1.33*	0.33
2008	-0.36	0.07	-0.33*	-0.23*	0.02	0.04	-0.05*	-0.10	-1.20	2.33	-0.67	0.33
2009	-0.42	0.50*	-0.37	-0.35	-0.16	0.07	-0.19*	0.17*	-1.01	-0.33	-5.33	3.00*
2010	-0.29[b]	0.33*	-0.31*	-0.17	-0.19	-0.06	-0.23*	-0.14	-3.24	0.67	-0.67	2.00
2011	-0.36	-0.18	-0.06*	-0.15	-0.02	0.04	-0.10*	0.16	-0.67*	9.33	-3.00	-0.67*
2012	0.09	0.82*	0.05	0.12	-0.05	0.27	-0.22	0.04	2.00*	2.33	0.67*	-0.67*
2013	-0.01	-0.51*	-0.36	0.37*	-0.30*	-0.06	-0.22*	-0.16	0.67*	0.33*	-1.00	0.67*
2014	-0.06	0.18	-0.33	0.46*	-0.12	1.02*	0.10	0.17	1.00*	-0.67	1.67*	-0.33
2015	-0.05[b]	0.70*	-0.55*	-0.05	-0.34	0.34*	-0.22	0.41	0.67*	-0.67*	1.67	2.33*
2016	-0.27	0.55	-0.02	0.43*	-0.28	-0.09	-0.15*	0.37	2.33[8]	-0.33	-1.00*	-1.33*
2017	0.10	0.75*	0.06	0.28	0.09	0.15	-0.04	-0.17	-0.33	0.00	0.00	0.00
2018	0.07	0.04	0.15	0.17*	-0.13	0.14	0.03	-0.08*	0.67	25.33	-0.33	1.33
2019	-0.14	0.66	0.37*	0.06	-0.36*	0.29	-0.18*	0.43*	-0.67	1.33	-0.67	0.33
TOTAL	34	31	30	26	34	31	30	26	34	31	30	26
Mean	-0.03	0.30	-0.01	0.05	-0.04	0.06	-0.13	0.02	0.63	2.97	-0.04	0.15
Decline	19, 3	3, 2	16, 7	9, 3	21, 8	12, 6	24, 12	14, 7	9, 2	6, 1	17, 6	11, 3
Increase	15, 3	28, 10	14, 5	17, 7	13, 4	19, 4	6, 0	12, 4	25, 13	25, 4	13, 5	15, 4

Continuation of APPENDIX S1: TABLE S1.

	GRASSHOPPERS MEDIUM[a]					SMALL[b]				SPIDERS[b]			
SITE	A	B	C	D		A	B	C	D	A	B	C	D
YEAR													
1985	-0.60*	……	……	……		0.04	……	……	……	-6.33*	……	……	……
1986	-0.29*	……	……	……		3.31*	……	……	……	1.33	……	……	……
1987	-0.08	……	……	……		0.65	……	……	……	0.00	……	……	……
1988	……	……	……	……		……	……	……	……	……	……	……	……
1989	-0.09	-0.31*	……	……		-3.63*	0.91	……	……	0.00	0.00	……	……
1990	-0.13	-1.23*	-0.12	……		0.87	-3.64	-7.35	……	0.00	0.33	1.33*	……
1991	-0.10*	0.04	0.11*	……		-4.88*	3.16	-1.78	……	0.67	0.33	1.33*	……
1992	0.04	-0.02	0.07	……		1.36*	-27.09*	-27.10*	……	0.67*	1.00*	1.00*	……
1993	-0.12*	-0.85*	-0.43	……		-11.08*	-3.11	-13.11	……	-0.67*	0.00	0.00	……
1994	0.23	-0.39*	-0.10	0.06		-4.87*	-0.37	-9.73	-1.75	0.00	1.00	0.33	0.33
1995	-0.05	0.11*	0.01	0.01		-7.14*	0.39	-6.37	-13.95	1.67	-0.67	3.67*	1.00*
1996	-0.07	0.08	-0.37*	-0.39*		0.88	-4.82	-5.27	-22.30	2.33	-0.67*	0.67	6.67*
1997	0.18*	-0.02	-0.32	-0.32		-3.23	-12.63	2.46	4.37	2.33*	0.00	0.33	8.67*
1998	0.29	0.00	0.39*	0.03		4.43	-2.93	4.65	-4.09	2.33	2.67	1.33*	21.00*
1999	0.20*	0.18	0.09	-0.09		-5.73	6.16	3.40	-9.40*	0.67	0.00	-2.00*	4.33*
2000	-0.01	-0.61*	-0.58	-0.21		-2.28	0.51	-0.33	-14.34*	0.33	1.00*	0.00	4.00*
2001	0.02	0.11	0.27	-0.29		1.53	-2.92*	-1.41	1.86	1.33	0.67	3.00*	19.33*
2002	-0.37*	0.06	-0.20*	-0.25		1.22	-2.42	-23.38	-9.70	1.00*	4.33*	0.67	26.00*
2003	0.00	1.06*	0.00	-0.81		1.92	7.47	-7.09	21.49	2.33*	7.67*	2.33	29.00*
2004	0.13	0.12	-0.12*	0.43*		-4.48	-3.61*	-4.49*	3.06	3.33*	1.00	0.67	2.67
2005	0.76*	-0.31	-0.60*	0.43		-2.33	2.33*	-0.96	1.22	1.67*	2.00*	0.67	8.67
2006	0.40	0.58*	-0.16*	1.00*		0.03	-2.30	-2.96*	-7.29*	0.67*	1.33*	0.33	3.33*
2007	0.33*	0.33	-0.10	0.14		1.81	0.67	1.00	2.33	0.33	0.67	3.67*	7.67*
2008	0.04	0.00	-0.04*	-0.12		-0.42	-0.33	-1.00*	0.88	1.67*	1.67	2.67*	13.33*
2009	-0.14	0.04	-0.17*	0.22		-5.37	0.00	0.33	-5.67	0.33	0.33	0.67	4.00*
2010	-0.06	-0.12	-0.21*	-0.12		-5.06*	0.00	-1.67*	-18.44*	0.33	0.67	1.00*	3.00*
2011	0.07	0.01	-0.07*	0.19		-0.67	-0.33	-3.33	-0.33	1.67*	0.67	0.33	23.00*
2012	-0.05	0.20	-0.25	-0.03		-2.33*	0.33	0.33	3.67	0.33	2.67*	0.00	3.00*
2013	-0.01	0.28	-0.19	-0.18*		-3.33	0.00	0.00	1.67	0.00	0.67	-0.33	0.00
2014	-0.15	1.03*	0.11	0.21		-11.33	-0.67	-2.00	5.67	-1.00*	-0.33	1.00	1.00*
2015	-0.34	0.34*	-0.23	0.50		0.00	0.00	-0.33	-2.00*	0.00	2.00*	0.67*	-0.67
2016	-0.37	-0.09	-0.14	1.43		-2.00	1.00	-1.33	0.67	-0.33	2.67*	0.00	1.00*
2017	0.10	0.13	-0.05	-0.13		1.00	-0.33	0.67*	-2.00	0.67	0.33	0.33	8.67*
2018	0.22	0.06	0.05	-0.05		-1.33	-1.00*	-1.00	-4.00	0.33	0.00	0.67*	1.00
2019	-0.36*	0.29	-0.17*	0.56*		1	2.33	-2.33	-0.67	1.33*	0.00	0.67*	3.00
TOTAL	34	31	30	26		34	31	30	26	34	31	30	26
Mean	-0.01	0.03	-0.12	0.09		-1.81	-1.56	-3.72	-2.45	0.63	1.12	0.90	7.81
Decline	20, 6	10, 5	21. 10	13, 2		19, 7	16, 4	22, 5	15, 5	4, 3	3, 1	2, 1	1, 0
Increase	14, 4	21, 5	9, 2	13, 3		15, 2	15, 1	8, 1	11, 0	30, 10	28, 9	28, 12	25, 19

APPENDIX S1: TABLE S2. Our results are compared with literature examples for plants and herbivores alone, and for system-wide responses (observed and probabilistic expectations, see text). p = plants, h = herbivores, D = decrease and I = increase with predator exclusion.

STUDY		TOP-DOWN Plant(p), Herbivore(h) (p = D, h = I)	Top-down (D – I) *[p X h]/100*	SYSTEM-WIDE I – D *[(1-p) X (1-h)]/100*	SYSTEM-WIDE I – I *[(1-p) X h]/100*	SYSTEM-WIDE D – D *[p X (1-h)]/100*	Stat., df, P
**I. Our study: plants, grasshoppers, predators**	**Observed[1]**	**38.8%, 41.3% (121)**	**13.2%**	**33.1%**	**28.1%**	**25.6%**	
	***Projected*[2]**		***16.0%***	***35.9%***	***25.3%***	***22.8%***	**I.1 vs. I.2: χ^2 = 0.66, 3, 0.88**
	**Only Sig.[3]**	**37.5% (40), 26.7% (45)**					
	***Projected*[2]**		***10.0%***	***45.8%***	***16.7%***	***27.5%***	**I.1 vs. I.4: χ^2 = 6.63, 3, 0.08**
**II. Other grasshopper and predator studies[a]:**							
**Both plants and grasshoppers measured**	**Observed[1]**	28.1%, 50.0% (32)	25.0%	40.00%	10.0%	25.00%	I.1 vs. II.1: χ^2 = 6.32, 3, 0.10
	***Projected*[2]**		*14.0%*	*36.0%*	*36.0%*	*14.0%*	I.1 vs. II.2: χ^2 = 5.27, 3, 0.15
**All studies**	**Observed[4]**	28.1% (32), 44.6% (52)					
	***Projected*[2]**		*12.5%*	*39.8%*	*32.1%*	*15.6%*	I.1 vs. II.4: χ^2 = 3.98, 3, 0.26
**III. Votes of predator and herbivore effects[b]:**	**Observed[4]**	48.2% (512), 30.0% (800)					
	***Projected*[2]**		*14.5%*	*36.3%*	*15.5%*	*33.7%*	
**IV. Meta-analyses converted to votes[5]:**							
**Review of terrestrial experiments[c]**							
**Both plants and herbivores studied**	**Observed[1]**	66.7%, 50.0% (36)	47.2%	30.6%	2.8%	19.4%	
	***Projected*[2]**		*33.4%*	*16.7 %*	*16.7%*	*33.4%*	
**All studies**	**Observed[4]**	58.3% (60), 50.0% (36)					
	***Projected*[2]**		*30.9%*	*19.2%*	*19.2%*	*30.9%*	
**Review of lake experiments[d]**							
**Both plants and herbivores studied**	**Observed[1]**	54.5%, 36.4% (11)	36.4%	45.5%	0%	18.2%	
	***Projected*[2]**		*19.8%*	*28.9%*	*16.6%*	*34.7%*	
**Single terrestrial trophic level results**	**Observed[4]**	38.8% (123)[e], 56.0% (40)[f]					
	***Projected*[2]**		*21.7%*	*26.9%*	*34.3%*	*17.1%*	
**V. Overall (II, III, IV) ---**							I.1 vs. V.1: plants - χ^2 = 0.17, 1, 0.68
**Both plants and herbivores studied**	**Observed[1]**	41.8%, 48.2% (79)					herbivores - χ^2 = 0.89, 1, 0.35
	***Projected*[2]**		*20.1%*	*30.1%*	*28.1%*	*21.7%*	I.1 vs. V.1 system: χ^2 = 1.91, 3, 0.59
**All studies**	**Observed[1]**	45.6 $\pm$ 5.5%, 43.4 $\pm$ 4.6%					I.1 vs. V.4: plants - t = 1.24, 4, 0.28
	***Projected*[2]**		*19.9 $\pm$ 3.2%*	*30.2 $\pm$ 3.6%*	*23.5 $\pm$ 4.0%*	*26.4 $\pm$ 4.2%*	herbivores - t = 0.45, 4, 0.67<br>I.1 vs. V.2 system: χ^2 = 2.31, 3, 0.51

Data employed:

1) Plants and herbivores simultaneously measured.
2) Projection based on independent probabilities of plant and herbivore responses (see text and column label).
3) Only observations when plants and herbivores simultaneously measured.
4) All plant and herbivore measures, whether measured simultaneously or not.
5) # of statistically significant top-down responses

Continuation of APPENDIX S1: TABLE S2.

<u>**References**</u>:
a) Belovsky & Slade 1993; Belovsky & Laws unpubl.; Bock et al. 1992; Branson 2005; Chase 1996; Danner & Joern 2003a, b; Floyd 1996; Fowler et al. 1991; Fuller & Joern 1996; Gardner & Thompson 1998; Joern & Rudd 1982; Joern 1986, 1992; Kajak et al. 1968; Laws & Joern 2015; Laws et al. 2009; Oedekoven & Joern 1998, 2000; Pitt 1999; Ritchie 2000; Saintignon & Kistner 2012; Schmitz 1993; 1994; 2010; Wineland et al. 2015
b) Sih et al. 1985, top-down effect of predators on herbivores (top-down effect of herbivores on plants); italics is estimate based on predator and herbivore effects
c) Schmitz et al. 2000
d) Brett and Goldman 1996
e) Shurin et al. 2002, Gruner et al. 2008
f) Holt et al. 2008

References for APPENDIX S1: TABLE S2.

Belovsky, Gary E., and Jennifer B. Slade. 1993. "The role of vertebrate and invertebrate predators in a grasshopper community." *Oikos* 68: 193-201.

Bock, Carl E., Jane H. Bock, and Michael C. Grant. 1992. "Effects of bird predation on grasshopper densities in an Arizona grassland." *Ecology* 73: 1706-1717.

Branson, David H. 2005. "Direct and indirect effects of avian predation on grasshopper communities in northern mixed-grass prairie." *Environmental Entomology* 34: 1114-1121.

Brett, Michael T., and Charles R. Goldman. 1996. "A meta-analysis of the freshwater trophic cascade." *Proceedings of the National Academy of Sciences USA* 93: 7723-7726. doi: 10,1073/pnas.93.15.7723.

Chase, Jonathan M. 1996. "Abiotic controls of trophic cascades in a simple grassland food chain." *Oikos* 77: 495-506.

Danner, Bradford J., and Anthony Joern. 2003a. "Stage-specific behavioral responses of *Ageneotettix deorum* (Orthoptera: Acrididae) in the presence of Lycosid spider predators." *Journal of Insect Behavior* 16: 453-464.

Danner, Bradford J., and Anthony Joern. 2003b. "Resource-mediated impact of spider predation risk on performance in the grasshopper *Ageneotettix deorum* (Orthoptera: Acrididae)." *Oecologia* 137: 352-359.

Floyd, Ted. 1996. "Top-down impacts on creosotebush herbivores in a spatially and temporally complex environment." *Ecology* 77: 1544-1555.

Fowler, Ada C., Richard L. Knight, T. Luke George, and Lowell C. McEwen. 1991. "Effects of avian predation on grasshopper populations in North Dakota grasslands." *Ecology* 72: 1775-1781.

Fuller, Rebecca C., and Anthony Joern. 1996. "Grasshopper susceptibility to predation in response to vegetation cover and patch area." *Journal of Orthopteran Research* 5: 175-183.

Gardner, Kevin T., and David C. Thompson. 1998. “Influence of avian predation on a grasshopper (Orthoptera: Acrididae) assemblage that feeds on Threadleaf Snakeweed.” *Environmental Entomology* 27: 110-116. doi: 10.1093/ee/27.1.110.

Gruner, Daniel S., Jennifer E. Smith, Eric W. Seabloom, Stuart A. Sandin, Jacqueline T. Ngai, Helmut Hillebrand, W. Stanley Harpole, James J. Elser, Elsa E. Cleland, Matthew E. S. Bracken, Elizabeth T. Borer, and Benjamin M. Bolker. 2008. “A cross-system synthesis of consumer and nutrient resource control on producer biomass.” *Ecology Letters* 11: 740-755. doi: 10.1111/j.1461-0248.2008.01192x.

Holt, Alison R., Zoe G. Davies, Claire Tyler, and Samantha C. Staddon. 2008. “Meta-analysis of the effects of predation on animal prey abundance: evidence from UK vertebrates.” *PLoS ONE* 3. e2400. ISSN 1932-6203.

Joern, Anthony. 1986. “Experimental study of avian predation on coexisting grasshopper populations (Orthoptera: Acrididae) in sandhills grassland.” *Oikos* 46: 243-249.

Joern, Anthony. 1992. “Variable impact of avian predation on grasshopper assemblies in sandhills grassland.” *Oikos* 64: 458-463.

Joern, Anthony, and Nathan T. Rudd. 1982. “Impact of predation by the robber fly *Proctacanthus milbertii* (Diptera: Asilidae) on grasshopper (Orthoptera: Acrididae) populations.” *Oecologia* 55: 42-46.

Kajak, Anna, Lucyna Andrzejewska, and Zdzislawa Wojcik. 1968. “The role of spiders in the decrease of damages caused by Acridoidea on meadows – experimental investigations.” *Ekologia Polska, Seria A* 16: 755-764.

Laws, Angela N., and Anthony Joern. 2015. “Predator-prey interactions are context dependent in a grassland plant-grasshopper-wolf spider food chain.” *Environmental Entomology* 44: 519-528.

Laws, Angela N., Therese C. Frauendorf, Jesus E. Gómez, and Isabel M. Algaze. 2009. “Predators mediate the effects of a fungal pathogen on prey: an experiment with grasshoppers, wolf spiders, and fungal pathogens.” *Ecological Entomology* 34: 702-708.

Oedekoven, Mark A., and Anthony Joern. 1998. “Stage-based mortality of grassland grasshoppers (Acrididae) from wandering spider (Lycosidae) predation.” *Acta Oecologia* 19: 507-515.

Oedekoven, Mark A., and Anthony Joern. 2000. “Plant quality and spider predation affects grasshoppers (Acrididae): food-quality-dependent compensatory mortality.” *Ecology* 81: 66-77.

Pitt, William C. 1999. “Effects of multiple vertebrate predators on grasshopper habitat selection: trade-offs due to predation risk, foraging, and thermoregulation.” *Evolutionary Ecology* 13: 499-515.

Ritchie, Mark E. 2000. "Nitrogen limitation and trophic vs. abiotic influences on insect herbivores in a temperate grassland." *Ecology* 81: 1601-1612.

Saintignon, Diana, and Erica Kistner. 2012. "The effects of Lycosoid spider density within trophic cascades." *The Journal of Undergraduate Research at Ohio State* 3: 23-28.

Schmitz, Oswald J. 1993. "Trophic exploitation in grassland food webs: simple models and a field experiment." *Oecologia* 93: 327-335.

Schmitz, Oswald J. 1994. "Resource edibility and trophic exploitation in an old field food web." *Proceedings of the National Academy of Sciences* 91: 5364–5367.

Schmitz, Oswald J. 2010. *Resolving Ecosystem Complexity.* Princeton: Princeton University Press.

Schmitz, Oswald J., Peter A. Hambäck, and Andrew P. Beckerman. 2000. "Trophic cascades in terrestrial systems: a review of the effects of carnivore removal on plants." *American Naturalist* 155: 141-153.

Shurin, Jonathan B., Elizabeth T. Borer, Eric W. Seabloom, Kurt Anderson, Carol A. Blanchette, Bernardo Broitman, Scott D. Cooper, and Benjamin S. Halpern. 2002. "A cross-ecosystem comparison of the strength of trophic cascades." *Ecology Letters* 5: 785-791.

Sih, Andrew, Philip Crowley, Mark McPeek, James Petranka, and Kevin Strohmeier. 1985. "Predation, competition, and prey communities: A review of field experiments." *Annual Review of Ecology and Systematics* 16: 269-311.

Wineland, Sean M., Erica J. Kistner, and Anthony Joern. 2015. "Non-consumptive interactions between grasshoppers (Orthoptera: Acrididae) and wolf spiders (Lycosidae) produce trophic cascades in an old-field ecosystem." *Journal of Orthoptera Research* 24: 41-46.
